# Lavandin cell extract enriched in rosmarinic acid attenuates inflammation via AMPK/Nrf2/HO-1 activation and IKK/IκB/NF-κB inhibition

**DOI:** 10.1186/s12870-026-09361-5

**Published:** 2026-06-27

**Authors:** Gyeongchan Jeon, Bo Ryeong Kim, Yu Jeong Jeong, Soyoung Kim, Jiyoung Lee, Ok Ran Lee, Hyung Jae Jeong, Jae Cheol Jeong, Cha Young Kim

**Affiliations:** 1 Biological Resource Center, Korea Research Institute of Bioscience Biotechnology (KRIBB), 181 Ipsin-gil, Jeongeup, 56212 Republic of Korea; 2 Department of Applied Plant Science, College of Agriculture and Life Sciences, Chonnam National University, Gwangju, 61186 Republic of Korea; 3 Functional Biomaterial Research Center, Korea Research Institute of Bioscience Biotechnology (KRIBB), Jeongeup, 56212 Republic of Korea

**Keywords:** Anti-inflammatory, Cell suspension cultures, Elicitation, Lavandin, Rosmarinic acid, Biomaterial

## Abstract

*Lavandula* species are rich in bioactive compounds, among which rosmarinic acid (RA) is a major phenolic known for potent antioxidant and anti-inflammatory activities. In this study, Lavandin (*Lavandula* × *intermedia*), a sterile hybrid widely cultivated for essential oil production, was used to develop RA-enriched cell extracts through plant cell culture technology combined with methyl jasmonate (MJ) elicitation. Lavandin cell suspension cultures accumulated higher levels of RA compared to those of *L. angustifolia*, and MJ treatment further enhanced RA biosynthesis, as confirmed by the upregulation of RA biosynthetic genes. Extracts derived from untreated and MJ-treated lavandin cell suspension cultures were assessed in LPS-stimulated RAW 264.7 macrophages. Both extracts were non-cytotoxic and significantly suppressed nitric oxide production, pro-inflammatory cytokines (TNF-α, IL-1β, IL-6), and the expression of iNOS and COX-2. Notably, the MJ-treated lavandin cell extract exhibited superior anti-inflammatory efficacy. Mechanistic analyses revealed that lavandin cell extract selectively inhibited the IKK/IκB/NF-κB pathway with minimal influence on MAPK pathways, while concurrently activating the AMPK/Nrf2/HO-1 axis. These findings demonstrate that MJ-elicited lavandin cell extract enriched in RA possesses strong anti-inflammatory activity and highlight its potential as a functional biomaterial for mitigating oxidative stress–related and inflammatory conditions.

**Supplementary Information:**

The online version contains supplementary material available at https://doi.org/10.1186/s12870-026-09361-5.

## Introduction

The genus *Lavandula* has been employed in traditional herbal medicine for centuries to treat restlessness, insomnia, agitation, and various nervous and inflammatory conditions, and these uses are supported by emerging evidence demonstrating antioxidant, anti-inflammatory, antimicrobial, anxiolytic, and analgesic activities of lavandin extracts and essential oils [1]. *Lavandula* species are widely recognized for their abundant production of phenolic compounds with diverse biological activities [2]. Among these compounds, rosmarinic acid (RA) has been extensively studied for its strong antioxidant, anti-inflammatory, and skin-protective properties, making it an attractive candidate for cosmetic and pharmaceutical applications [3]. In our previous study, we established cell suspension cultures of *L. angustifolia* and demonstrated that methyl jasmonate (MJ) elicitation significantly enhanced RA biosynthesis [4]. Furthermore, the resulting RA-enriched extract exhibited multiple bioactivities, including protection against UV-induced oxidative stress, suppression of pro-inflammatory cytokines, and stimulation of skin-related functions [5]. These findings underscore the feasibility of plant cell cultures as a platform for producing multifunctional bioactive materials.

Building on these results, we turned our attention to the potential of lavandin (*Lavandula x intermedia*), a natural hybrid between *L. angustifolia* and *L. latifolia*. Lavandin is one of the most widely cultivated *Lavandula* species due to its abundant biomass, superior essential oil yield, and distinct metabolite profile compared with *L. angustifolia* [1, 6]. However, as a sterile hybrid, lavandin cannot be propagated by seeds and is maintained exclusively through vegetative cuttings, which limits its genetic diversity and breeding potential [7]. This reproductive constraint further underscores the importance of alternative approaches, such as plant cell culture techniques, for exploring and utilizing its bioactive metabolites. Although lavandin is extensively cultivated for its essential oil production, scientific investigations of this hybrid remain relatively limited [6], and plant cell culture-based studies are particularly scarce compared with those of lavender. Given the pharmacological importance of RA, further investigation of lavandin cell cultures as a potential source of this compound is warranted.

Extensive investigations, both in vitro and in vivo, have shown that RA—a predominant phenolic constituent of lavender—possesses strong anti-inflammatory activity [8]. These findings support the medicinal potential of lavender and its derivatives [9]. Given these findings, we hypothesized that lavandin cell extract enriched in RA, produced using a cell culture system with MJ elicitation, could serve as a potent anti-inflammatory agent. To verify this, we designed an in vitro study using LPS-stimulated RAW 264.7 macrophages, a widely used model for evaluating the anti-inflammatory activity of natural compounds [10]. LPS treatment provokes an inflammatory cascade that includes the release of prostaglandin E2 (PGE2), nitric oxide (NO), and multiple cytokines, driven by inducible enzymes like COX-2 and iNOS [11].

Inflammatory responses in macrophages are primarily governed by NF-κB and MAPK signaling pathways, which regulate the production of iNOS, COX-2, and pro-inflammatory cytokines. In parallel, antioxidant defenses are modulated by the AMPK/Nrf2/HO-1 axis, a key pathway that counteracts oxidative and inflammatory stress [12–15]. These mechanisms collectively provide the biological context for evaluating the anti-inflammatory potential of RA-enriched lavandin cell extract [16].

Plant cell and tissue cultures offer unique advantages for producing valuable phytochemicals under controlled conditions, independent of environmental or seasonal constraints [17, 18]. Moreover, elicitor-mediated enhancement of secondary metabolites, particularly the use of MJ, is well established for activating phenylpropanoid metabolism and enhancing RA accumulation [4, 19]. However, systematic evaluation of MJ effects on lavandin cell cultures and their functional bioactivities has been rarely reported. To our knowledge, this is the first to reveal the impact of elicitation on lavandin cell cultures, its contribution to RA biosynthesis, and its impact on anti-inflammatory activity. Specifically, we established callus and cell suspension cultures of *L. intermedia*, optimized MJ elicitation for RA production, and analyzed the expression of RA biosynthetic genes. We also examined the anti-inflammatory properties of lavandin cell extract in LPS-stimulated RAW 264.7 macrophages. Our findings demonstrate that lavandin cell extract exerts its anti-inflammatory effects by activating the AMPK/Nrf2/HO-1 pathway while strongly suppressing the IKK/IκB/NF-κB pathway. Collectively, this work extends previous findings in *L. angustifolia* by developing RA-enriched lavandin cell extract through MJ elicitation and confirming its enhanced bioactivities, underscoring its potential as a sustainable source of RA-enriched functional biomaterials.

## Materials and methods

### Plant materials and callus induction

*Lavandula × intermedia* Emeric ex Loisel. (Lamiaceae) plants were obtained from a commercial herb farm (Herbone, Jeongeup, Republic of Korea), a certified supplier, and used as donor material for establishing plant cell culture. The plant material was authenticated based on supplier documentation and phenotypic characteristics. As the study was conducted using plant cell cultures derived from cultivated donor plants rather than field-collected specimens, no voucher specimen was deposited in a public herbarium. Explants (5–8 mm fragments) were cultured on half-strength Murashige and Skoog (MS) basal medium (2.2 g/L) supplemented with 30 g/L sucrose, 1 mg/L 6-benzylaminopurine (BAP), 0.3 g/L 2,4-dichlorophenoxyacetic acid (2,4-D), 0.4 mg/L thiamine, 6 g/L myo-inositol, and 4 g/L gelrite (referred to as 1/2MS1B0.3D0.6myo medium). The pH of the medium was adjusted to 5.8 before autoclaving. Cultures were maintained in the dark at 25℃, and subcultured every two weeks. All media constituents were obtained from Duchefa Biochemie (Haarlem, The Netherlands).

### Cell suspension cultures and cell growth analysis

Suspension cultures were initiated from stem-derived calli of *L. intermedia* and maintained in liquid 1/2MS1B0.3D0.6myo medium. Briefly, 20 mL of medium in a 125 mL Erlenmeyer flask was inoculated with 2 g of callus (10% *w/v*) and incubated at 25 ± 1 °C under continuous agitation (90 rpm) in the dark. Cell growth was monitored by measuring the dry weight (DW) of cells every two days for up to 22 days.

### Elicitation and sample collection

Seven-day-old suspension cultures were treated for 5 days with various elicitors—methyl jasmonate (MJ, 100 µM), salicylic acid (100 µM), ethephon (300 µM), or abscisic acid (ABA, 300 µM)—as described previously [4, 20]. A 0.1% ethanol treatment served as a control. For optimization, MJ was applied to 7-day-old pre-culture at final concentrations ranging from 0 to 300 µM, and the cells were harvested after 5 days. Time-course experiments were performed by treating cultures with 100 µM MJ and collecting samples at 0, 1, 3, 5, 7, 9, 12, and 15 days post-treatment. All samples were frozen in liquid nitrogen, lyophilized, and processed for RA quantification.

### High-performance liquid chromatography (HPLC) analysis of RA

RA was extracted and quantified according to the method of [4]. In brief, 0.1 g of lyophilized sample was sonicated in 10 mL of 80% methanol, filtered through a 0.2 μm PTFE membrane, and analyzed using HPLC equipped with a UV detector set at 340 nm. The mobile phase consisted of water (solvent A) and acetonitrile (Honeywell, USA) containing 0.02% acetic acid (solvent B) at a flow rate of 0.8 mL/min. RA was identified and quantified using a commercial standard (Sigma-Aldrich, USA).

### Preparation of cell extracts

Lavandin suspension-cultured cells treated with or without 100 µM MJ for 7 days were harvested, dried, and extracted with 80% methanol using ultrasonic treatment for 30 min. Extracts were filtered, dried, and re-dissolved in dimethyl sulfoxide (DMSO). The resulting samples were designated as lavandin cell extracts from untreated cells (LIC-CK) or MJ-treated cells (LIC-MJ).

### DPPH radical scavenging assay

DPPH radical scavenging activity was evaluated by mixing various concentrations of the samples with a DPPH solution (Sigma-Aldrich) and incubating the mixture in the dark for 30 min. The absorbance was measured at 517 nm using a UV–Vis microplate spectrophotometer (Multiskan SkyHigh; Thermo Fisher Scientific, Waltham, MA, USA). A solvent blank and L-ascorbic acid (AsA; Sigma Aldrich, USA) were used as the negative and positive controls, respectively. Inhibition percentage was calculated as: Inhibition (%) = 100 x [1 - (Absorbance of sample / Absorbance of blank)]. IC₅₀ values were determined by linear interpolation between the two concentrations bracketing 50% scavenging activity. All experiments were performed in triplicate.

### Reverse transcription quantitative PCR (RT-qPCR) analysis

#### RA biosynthesis-related gene expression

To examine RA biosynthesis, lavandin cells were harvested at 0, 6, 12, and 24 h after MJ treatment. RNA extraction, cDNA synthesis, and RT-qPCR were performed as previously described [4]. Tubulin was used as an internal control, and relative expression was calculated using the 2^−△△CT^ method [21]. Primer sequences are provided in Table S1.

#### Inflammatory gene expression in RAW 264.7 cells

RAW 264.7 murine macrophages were seeded at 5 × 10^6^ cells/dish, serum-starved for 12 h, pretreated with LIC-CK or LIC-MJ for 1 h, and then stimulated with LPS (0.5 µg/mL) for 4 h. RNA isolation, cDNA synthesis, and RT-qPCR were performed following the manufacturer’s protocols (GeneAll, GeNet Bio, South Korea). Primer sequences are listed in Table S1. 

### Mammalian cell culture

RAW 264.7 macrophages (KCLB No. 470071) were obtained from the Korean Cell Line Bank and cultured in Dulbecco’s Modified Eagle Medium (DMEM; Gibco, USA) supplemented with 10% fetal bovine serum (FBS; Gibco, USA) and 100 U/mL penicillin–streptomycin at 37 °C in 5% CO₂.

### Cell viability assay

Cell viability was assessed using the EZ-Cytox assay kit (DoGenBio, South Korea). Briefly, RAW 264.7 cells were seeded in 96-well plates (5 × 10^4^ cells/well), serum-starved for 12 h, and treated with LIC-CK or LIC-MJ (25–200 µg/mL) for 24 h. Cell viability was determined by measuring absorbance at 450 nm 2 h after incubation with EZ-Cytox reagent.

### Measurement of NO, TNF-α, IL-1β and IL-6 cytokine production

RAW 264.7 cells (2 × 10^5^cells/well) were pretreated with LIC-CK or LIC-MJ for 1 h, then stimulated with LPS (0.5 µg/mL) for 24 h. Nitric oxide (NO) levels in the culture medium were quantified using the Griess reagent (Sigma-Aldrich, USA) according to the manufacturer’s instructions. Cytokine (TNF-α, IL-1β, IL-6) concentrations were determined using DuoSet ELISA kits (R&D Systems, USA) following the manufacturer’s protocol.

### Protein isolation and Western blotting analysis

RAW 264.7 cells (5 × 10^6^ cells/dish) were pretreated with LIC-CK or LIC-MJ for 1 h, stimulated with LPS (0.5 µg/mL) for 15 min–12 h, and harvested for protein extraction. Total proteins were extracted using PRO-PREP™ solution (iNtRON Biotechnology, South Korea), and cytoplasmic and nuclear fractions were obtained with NE-PER™ reagents (Thermo Scientific, USA). Protein concentrations were determined by BCA assay, and equal amounts of protein (10–20 µg) were subjected to SDS-PAGE and transferred for immunoblotting. Primary and secondary antibodies used in this study are listed in Table S2. PM2700 ExcelBand™ 3-color Broad Range Protein Marker (SMOBIO, South Korea) was used as a molecular weight marker.

## Results

### Callus induction and growth characteristics in *L. intermedia* cell suspension cultures

**Fig. 1:**
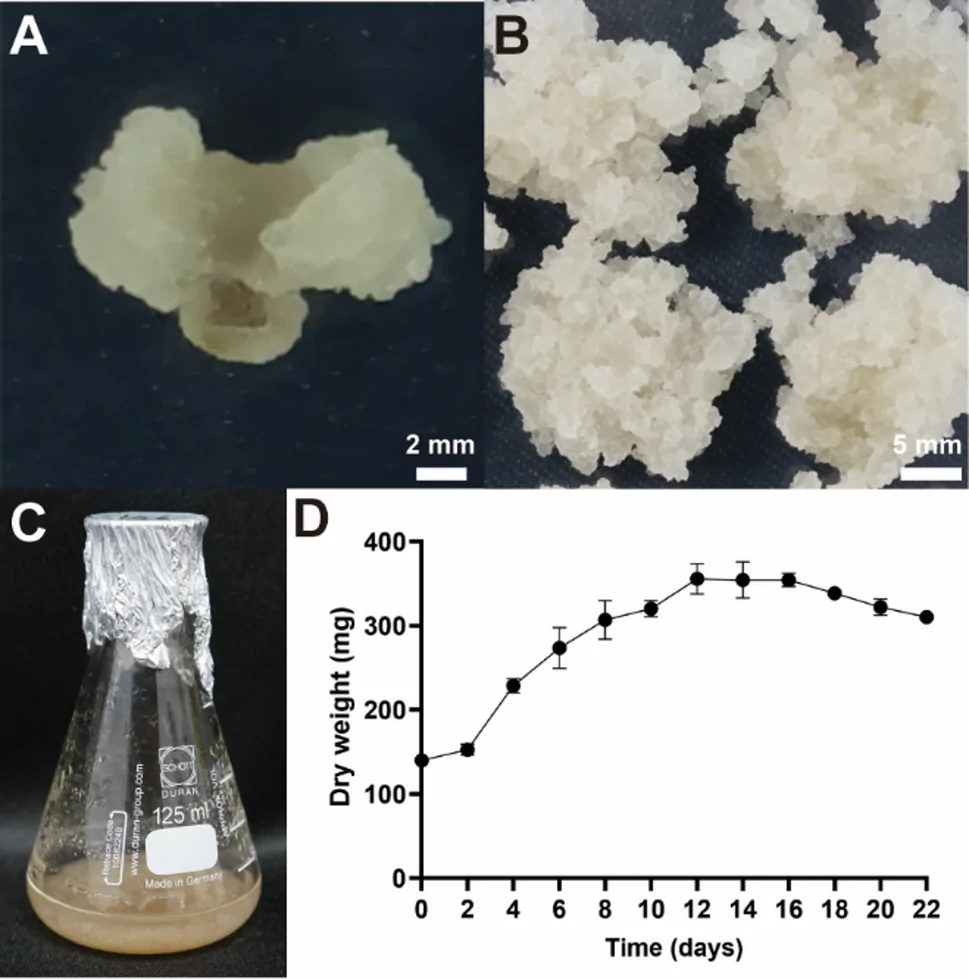
Callus induction and growth of lavandin callus and cell suspension cultures. (**A**) Callus formed from lavandin stem explants after 10 days of culture. (**B**) Friable callus obtained after subculture. (**C**) Cell suspension cultures established from lavandin callus. (**D**) Growth curve of *L. intermedia* cell suspension cultures based on dry weight during a 22-day culture period. Data are means ± SD (*n* = 3)

Callus induction was initiated from stem explants of *L. intermedia* cultivated on 1/2MS1B0.3D0.6myo medium, with visible callus formation occurring within 10 days (Fig. 1A). The calli were light beige, friable, and exhibited vigorous growth, allowing subculture at 2–3 week intervals (Fig. 1B). Cell suspension cultures exhibited a growth profile characterized by a remarkable increase in biomass, reaching 356 mg DW on day 12 before slowing and entering a stationary phase (Fig. 1C, D).

### Effects and optimization of elicitation on RA production in *L. intermedia* cell suspension cultures

**Fig. 2:**
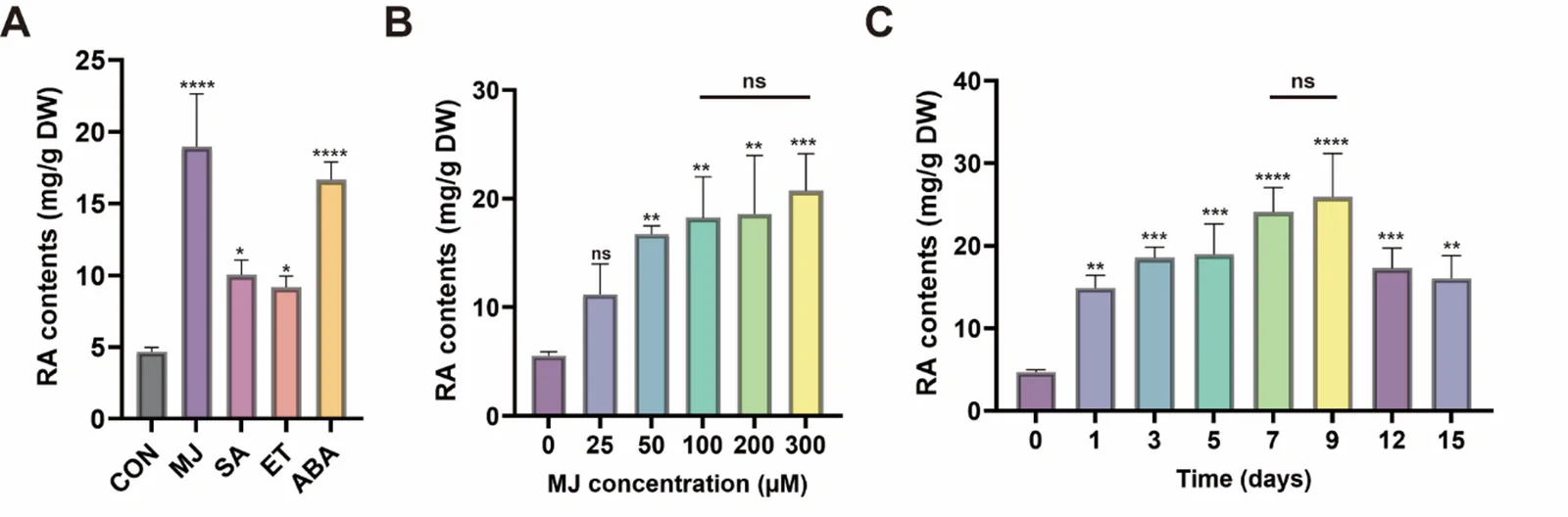
Effects and optimization of elicitation on RA production in lavandin cell suspension cultures. (**A**) Effects of different elicitors, including methyl jasmonate (MJ), salicylic acid (SA), ethephon (ET), and abscisic acid (ABA), on RA content after 5 days of treatment. (**B**) Effects of MJ concentration (0-300 µM) on RA content after 5 days of treatment. (**C**) Time course of RA accumulation in cell cultures treated with 100 µM MJ. Data are means ± SD (*n* = 3). Statistical significance is indicated relative to the control. ns, not significant; *, *p* < 0.05; **, *p* < 0.01; ***, *p* < 0.001; ****, *p* < 0.0001

To identify the most effective elicitor for stimulating RA biosynthesis, cell suspension cultures of *L. intermedia* were treated with MJ, SA, ET, and ABA for 5 days. HPLC analysis revealed that MJ treatment induced the highest RA content (19 mg/g DW) (Fig. 2A). Further optimization was performed by applying concentrations from 0 to 300 µM MJ for 5 days. RA accumulation exhibited a concentration-dependent increase, ranging from 5.5 mg/g DW in untreated cultures to 20.7 mg/g DW at 300 µM MJ. Although the highest RA content was observed at 300 µM, RA levels plateaued above 100 µM; therefore, 100 µM was considered the optimal concentration for RA in *L. intermedia* cell suspension cultures (Fig. 2B). A time-course analysis at the optimal MJ concentration of 100 µM revealed a progressive increase in RA content during the early culture phase, peaking at 27.7 mg/g DW on day 9, followed by a decline with prolonged incubation. These findings indicate that extending the culture period beyond 9 days does not further improve RA yield (Fig. 2C). A comparative analysis with our previous study on *L. angustifolia* cell suspension cultures under identical elicitation conditions (Figure S1) revealed that *L. intermedia* consistently produced higher RA levels across all treatments. In addition, *L. intermedia* exhibited a distinct production pattern, with RA levels peaking later and declining more gradually, suggesting a greater capacity and prolonged biosynthetic activity for RA production compared with *L. angustifolia*.

### MJ-induced expression of RA biosynthetic genes in *L. intermedia* cell suspension cultures

**Fig. 3:**
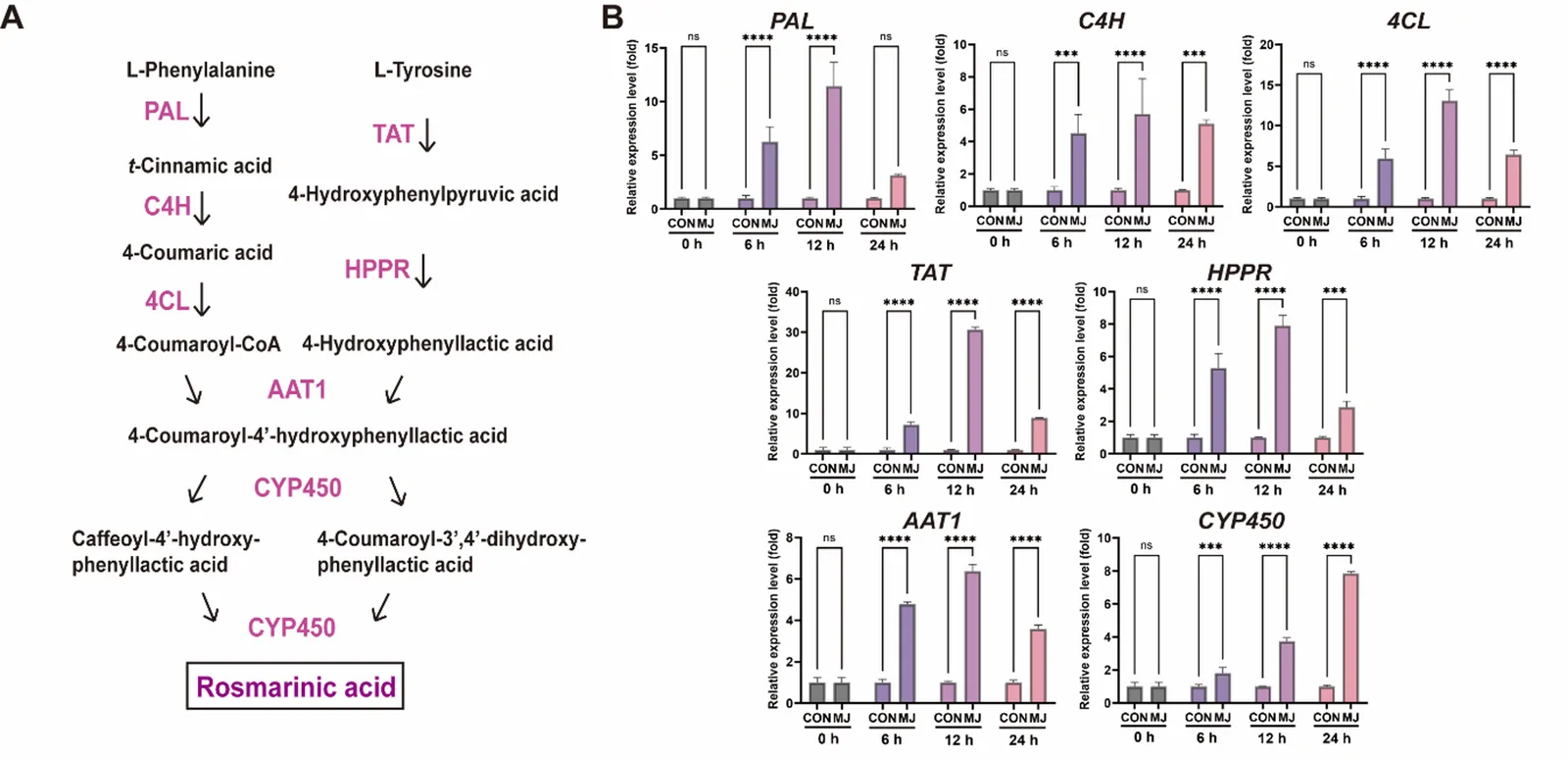
Effect of MJ treatment on the expression of RA biosynthetic genes in lavandin cell suspension cultures. (**A**) Proposed RA biosynthetic pathway in lavandin showing key enzymes. (**B**) Relative expression levels of *PAL*, *C4H*, *4CL*, *TAT*, *HPPR*, *AAT1*, and *CYP450* in control (CON) and MJ-treated (MJ) cultures at 0, 6, 12, and 24 h after treatment, determined by RT-qPCR. Data are means ± SD (*n* = 3). Statistical significance is indicated relative to the control. ns, not significant; ***, *p* < 0.001; ****, *p* < 0.0001

RA biosynthesis begins with L-phenylalanine and L-tyrosine and proceeds through a multistep enzymatic pathway, ultimately leading to RA formation via the action of rosmarinic acid synthase (RAS/AAT1) and cytochrome P450 monooxygenases [4, 22]. Based on our previous findings that RA biosynthesis is transcriptionally regulated in *L. angustifolia* cell suspension cultures [4], we examined the transcriptional response of key structural genes of *PAL*, *C4H*, *4CL*, *TAT*, *HPPR*, *AAT1*, and *CYP450* to MJ treatment in *L. intermedia* cell suspension cultures using RT-qPCR (Fig. 3). Consistent with our previous findings, most genes showed a gradual increase in expression within 6 h, peaked at 12 h post-treatment, and declined thereafter. At 12 h, transcript levels of *PAL*, *C4H*, *4CL*, *TAT*, *HPPR*, and *AAT1* increased by approximately 11.4-, 5.7-, 13.1-, 30.6-, 7.9-, and 6.4-fold, respectively, relative to the control. Notably, *AAT1*, which encodes rosmarinic acid synthase (RAS)–a key enzyme in RA biosynthesis [4, 22, 23]–was markedly upregulated by MJ treatment, underscoring its pivotal role in RA accumulation in *L. intermedia* cell suspension cultures. In contrast, *CYP450* expression peaked at 24 h with a 7.8-fold induction, followed by a decline to 3.5-fold at 48 h (data not shown). Collectively, these results demonstrate that MJ treatment enhances RA biosynthesis by coordinately activating RA biosynthetic genes in *L. intermedia* cell suspension cultures.

### MJ treatment enhances DPPH radical scavenging acticity

*L. intermedia* cell culture extracts were prepared without MJ elicitation (LIC-CK) or with MJ elicitation (LIC-MJ), as described in the Materials and Methods. The DPPH radical scavenging activities of both LIC-CK and LIC-MJ increased in a concentration-dependent manner, and LIC-MJ consistently exhibited higher antioxidant activity than LIC-CK. The IC_50_ values were 88.1 ± 11.6 µg/mL for LIC-CK and 54.3 ± 8.2 µg/mL for LIC-MJ, indicating that MJ elicitation significantly enhanced the antioxidant capacity of the extract (Table 1). The IC_50_ value of AsA was expressed as < 7.81 µg/mL.

**Table 1:** IC₅₀ values of DPPH radical scavenging activity

Sample	IC_50_ (µg/mL)
LIC-CK	88.1 ± 11.6
LIC-MJ	54.3 ± 8.2

### Lavandin cell extract reduced LPS-induced inflammatory responses in RAW 264.7 cells

**Fig. 4:**
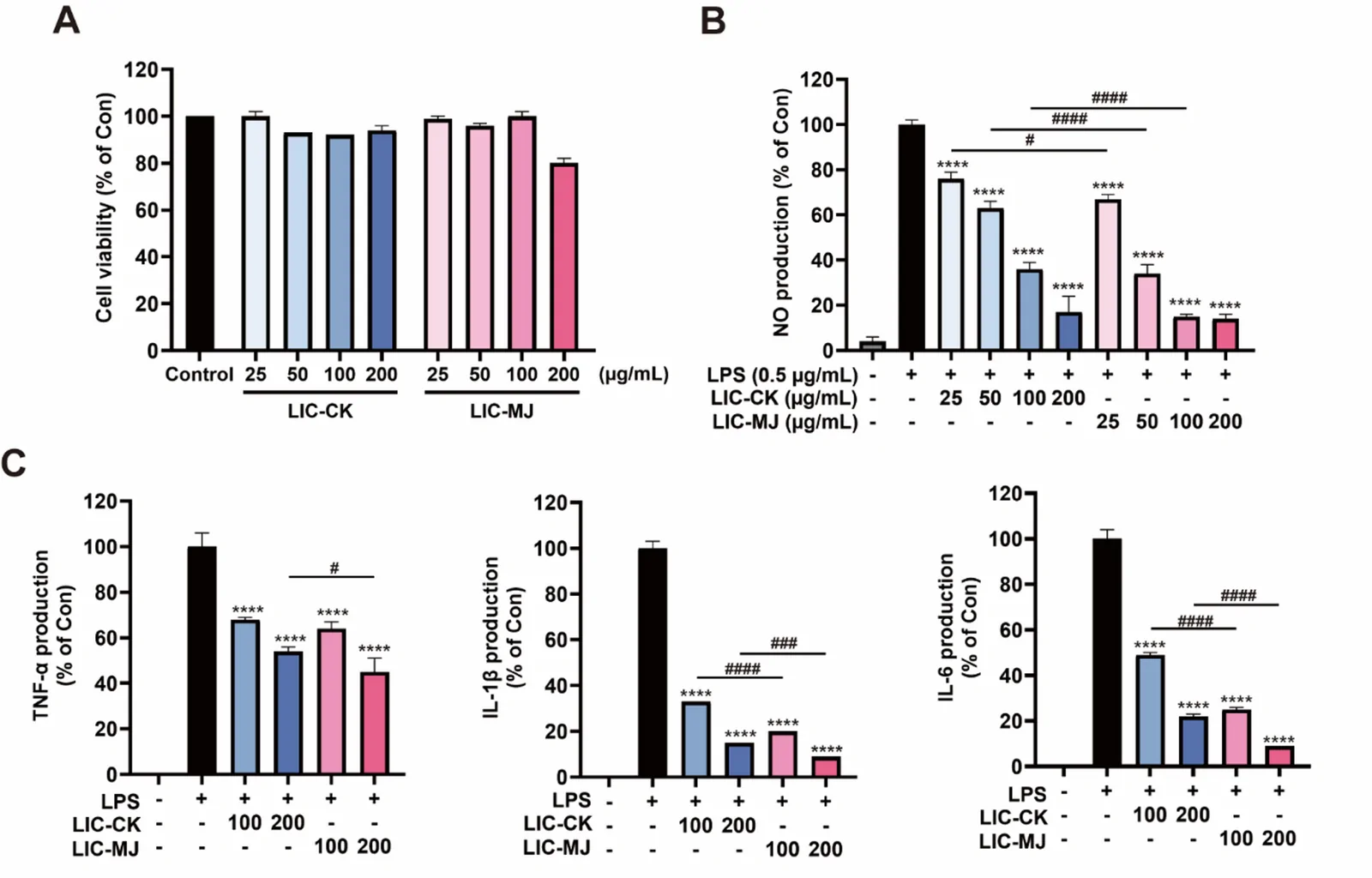
Cytotoxicity and anti-inflammatory effects of lavandin cell extracts in RAW 264.7 cells. (**A**) Cytotoxicity of LIC-CK and LIC-MJ was assessed using the EZ-Cytox cell viability assay kit after 24 h of treatment. (**B**, **C**) For NO and cytokine production analysis, RAW 264.7 cells were pretreated with LIC-CK or LIC-MJ for 1 h, followed by stimulation with 0.5 µg/mL LPS for 24 h. (**B**) NO production in culture supernatants was quantified using Griess reagent. (**C**) TNF-α, IL-1β, and IL-6 concentrations were measured using ELISA kits. Values are expressed relative to the LPS-treated control. Data are means ± SD (*n* = 3). Statistical significance is indicated relative to the control. ****, *p* < 0.0001 vs. LPS group. #, *p* < 0.05 vs. LIC-CK group

The anti-inflammatory effects of LIC-CK and LIC-MJ were evaluated in LPS-stimulated RAW 264.7 macrophages. Cytotoxicity testing showed that both LIC-CK and LIC-MJ extracts were non-toxic to RAW 264.7 macrophages at concentrations up to 200 µg/mL (Fig. 4A). Compared with the untreated control, pretreatment with either extract significantly reduced LPS-induced NO production, with LIC-MJ showing stronger inhibition (85%) than LIC-CK (64%) at 100 µg/mL (Fig. 4B). Specifically, NO levels were 0.10 µM in the -LPS group and 2.69 µM in the control (+ LPS) group. In LIC-CK-treated cells, NO production decreased to 2.06, 1.69, 0.98, and 0.46 µM at 25, 50, 100, and 200 µg/mL, respectively, whereas in LIC-MJ-treated cells, NO levels were further reduced to 1.80, 0.92, 0.41, and 0.37 µM at the corresponding concentrations.

Additionally, the effects of LIC-CK and LIC-MJ extracts on the inflammatory cytokines were examined by ELISA and RT-qPCR. ELISA showed that both extracts suppressed the production of pro-inflammatory cytokines (TNF-α, IL-1β, and IL-6) in LPS-stimulated RAW 264.7 cells (Fig. 4C). Specifically, TNF-α levels increased from 1.8 pg/mL in the -LPS group to 1083.3 pg/mL in the control (+ LPS) group, and were reduced to 739.7 and 584.4 pg/mL by LIC-CK (100 and 200 µg/mL), and to 688.8 and 488.9 pg/mL by LIC-MJ (100 and 200 µg/mL) at the corresponding concentrations. Similarly, IL-1β levels increased to 117.3 pg/mL in the control group but decreased to 38.3 and 17.5 pg/mL with LIC-CK, and to 23.6 and 10.7 pg/mL with LIC-MJ treatment. IL-6 levels were elevated from 0.7 pg/mL to 1101.0 pg/mL following LPS stimulation and were reduced to 544.1 and 241.3 pg/mL by LIC-CK, and to 276.6 and 101.8 pg/mL by LIC-MJ.

**Fig. 5:**
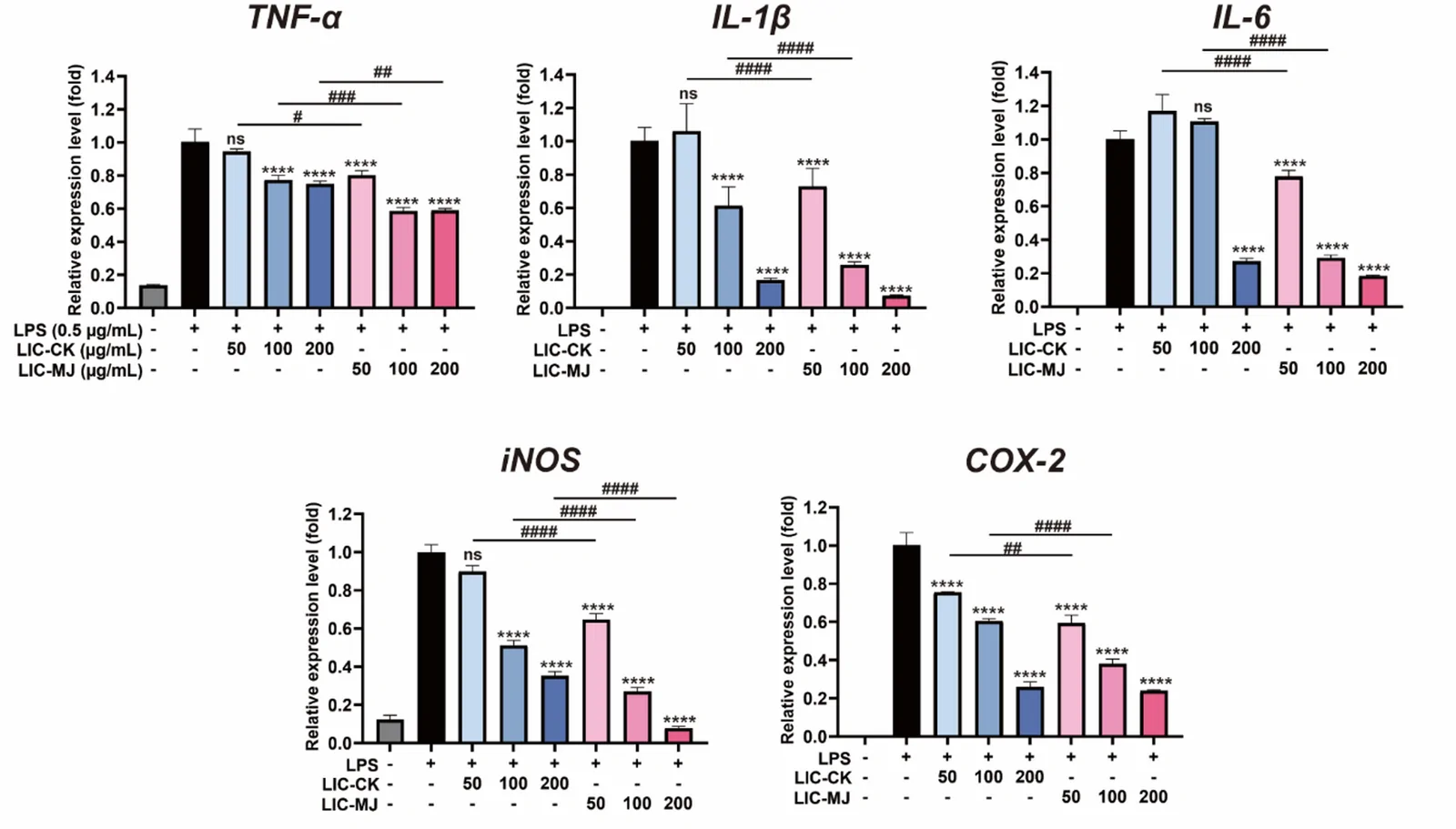
Effects of lavandin cell extracts on inflammatory gene expression in LPS-stimulated RAW 264.7 cells. RAW 264.7 cells were pretreated with LIC-CK or LIC-MJ for 1 h, followed by stimulation with 0.5 µg/mL LPS for 4 h. mRNA expression levels of *TNF-α*, *IL-1β*, *IL-6*, *iNOS*, and *COX-2* were quantified by RT-qPCR. Data are expressed relative to the LPS-treated control and are means ± SD (*n* = 3). Statistical significance is indicated relative to the control. ns, not significant; ****, *p* < 0.0001 vs. LPS group. #, *p* < 0.05; ##, *p* < 0.01; ###, *p* < 0.001; ####, *p* < 0.0001 vs. LIC-CK group

Consistently, RT-qPCR analysis revealed reduced mRNA expression of these cytokines, with stronger inhibition observed for interleukins (Fig. 5). Specifically, relative to the control, TNF-α expression decreased to 0.77- and 0.75-fold with LIC-CK (100 and 200 µg/mL) and to 0.58- and 0.59-fold with LIC-MJ (100 and 200 µg/mL), respectively. IL-1β expression was markedly reduced to 0.61- and 0.17-fold with LIC-CK and to 0.26- and 0.07-fold with LIC-MJ. Similarly, IL-6 expression decreased to 1.11- and 0.27-fold with LIC-CK and to 0.29- and 0.19-fold with LIC-MJ. Consistent with the NO data, LIC-MJ showed greater suppression of cytokine production than LIC-CK, particularly for IL-1β and IL-6, indicating its superior anti-inflammatory potential.

**Fig. 6:**
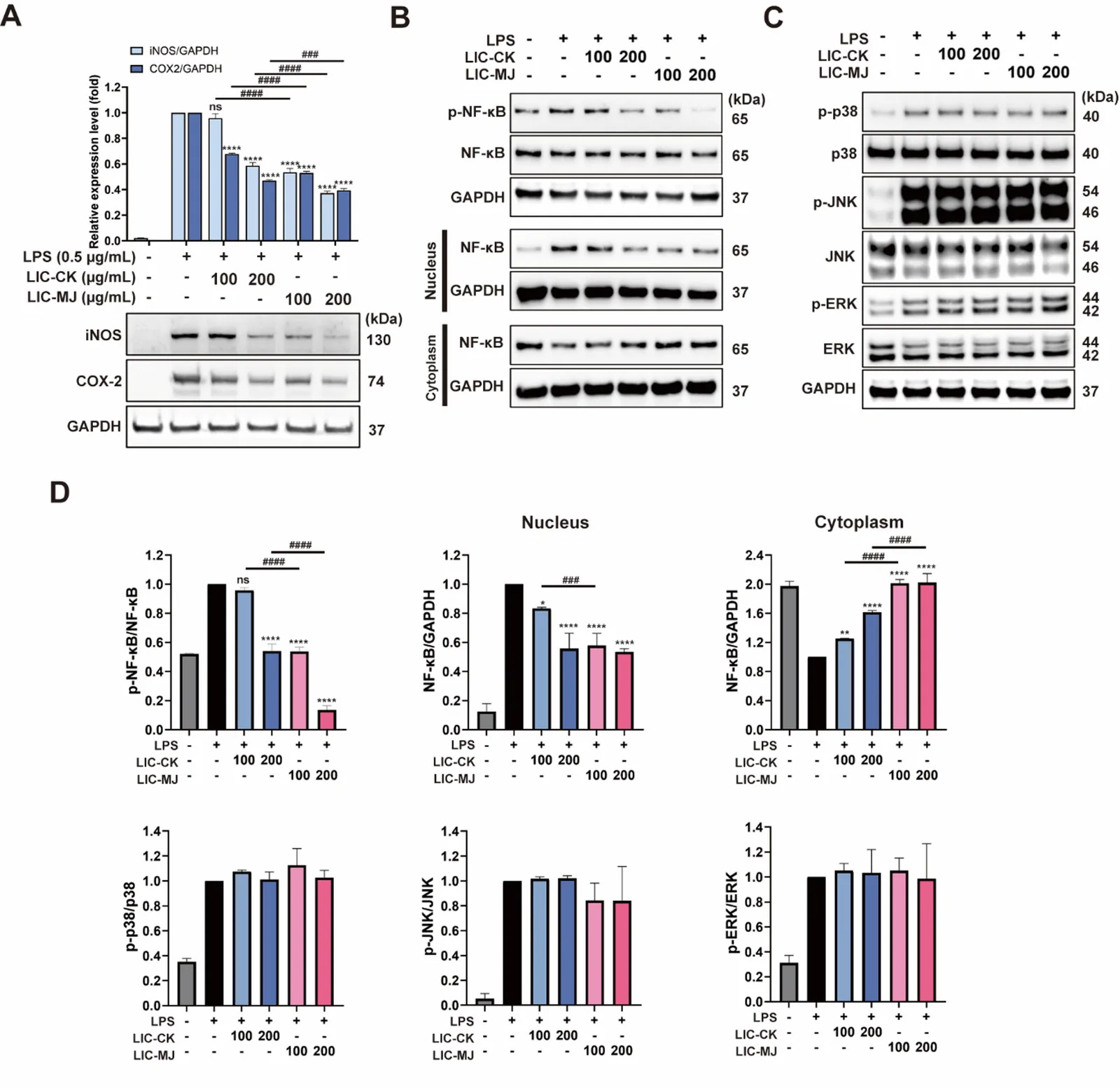
Inhibitory effects of lavandin cell extracts on iNOS and COX-2 expression and NF-κB/MAPK activation in LPS-stimulated RAW 264.7 cells. (**A**) RAW 264.7 cells were pretreated with LIC-CK or LIC-MJ for 1 h and then stimulated with 0.5 µg/mL LPS for 12 h. iNOS and COX-2 protein levels were analyzed by Western blotting. (**B**, **C**) RAW 264.7 cells were pretreated with LIC-CK or LIC-MJ for 1 h and then stimulated with 0.5 µg/mL LPS for 30 min. (**B**) Phosphorylated NF-κB (p-NF-κB), total NF-κB in whole-cell lysates, and the subcellular distribution of NF-κB in cytoplasmic and nuclear fractions were analyzed by Western blotting. (**C**) MAPK pathway proteins (p-p38, p38, p-JNK, JNK, p-ERK1/2 and ERK1/2) were detected by Western blotting. (**D**) Densitometric analysis of blots shown in panels B and C. Values are expressed relative to the LPS-treated control. Data are means ± SD (*n* = 3). Statistical significance is indicated relative to the control. ns, not significant; *, *p* < 0.05; **, *p* < 0.01; ***, *p* < 0.001; ****, *p* < 0.0001 vs. LPS group. ###, *p* < 0.001; ####, *p* < 0.0001 vs. LIC-CK group

To further investigate the mechanism of action, the effects of LIC-CK and LIC-MJ on *iNOS* and *COX-2* expression were examined by RT-qPCR and Western blotting. iNOS and COX-2, enzymes responsible for the synthesis of nitric oxide and prostaglandins, respectively, are critical mediators of inflammatory responses and common targets of anti-inflammatory drugs [11]. RT-qPCR analysis revealed that both LIC-CK and LIC-MJ extracts effectively reduced the expression of *iNOS* and *COX-2* (Fig. 5). Specifically, iNOS expression decreased to 0.51- and 0.35-fold with LIC-CK (100 and 200 µg/mL) and to 0.27- and 0.08-fold with LIC-MJ (100 and 200 µg/mL), respectively. Similarly, COX-2 expression was reduced to 0.61- and 0.26-fold with LIC-CK and to 0.38- and 0.24-fold with LIC-MJ. Western blotting further verified that both LIC-CK and LIC-MJ also suppressed the protein levels of iNOS and COX-2 (Fig. 6A). Specifically, relative to the control, iNOS protein levels decreased to 0.96- and 0.59-fold with LIC-CK (100 and 200 µg/mL) and to 0.53- and 0.37-fold with LIC-MJ (100 and 200 µg/mL), respectively. Similarly, COX-2 protein expression was reduced to 0.68- and 0.47-fold with LIC-CK and to 0.53- and 0.39-fold with LIC-MJ, with LIC-MJ showing greater suppression. Overall, the anti-inflammatory effects of lavandin cell extracts were dose-dependent, with LIC-MJ exhibiting superior efficacy than LIC-CK.

### Lavandin cell extracts inhibited the NF-κB signaling pathway and promoted AMPK/Nrf2/HO-1 signaling pathways

To investigate the anti-inflammatory mechanism of lavandin cell extracts, we examined the effects of LIC-CK and LIC-MJ extracts on NF-κB and MAPK signaling by Western blotting. As shown in Fig. 6B, both LIC-CK and LIC-MJ markedly reduced NF-κB activation and nuclear translocation, whereas no significant changes were detected in MAPK-related proteins, including p-p38, p38, p-JNK, JNK, p-ERK1/2, and ERK1/2 (Fig. 6C). Phosphorylation of NF-κB decreased to 0.96- and 0.54-fold with LIC-CK (100 and 200 µg/mL) and to 0.54- and 0.14-fold with LIC-MJ (100 and 200 µg/mL), respectively. Consistently, nuclear NF-κB levels were reduced to 0.83- and 0.56-fold with LIC-CK and to 0.58- and 0.53-fold with LIC-MJ. These findings confirm that lavandin cell extracts significantly suppressed NF-κB signaling during LPS-induced inflammatory responses.

**Fig. 7:**
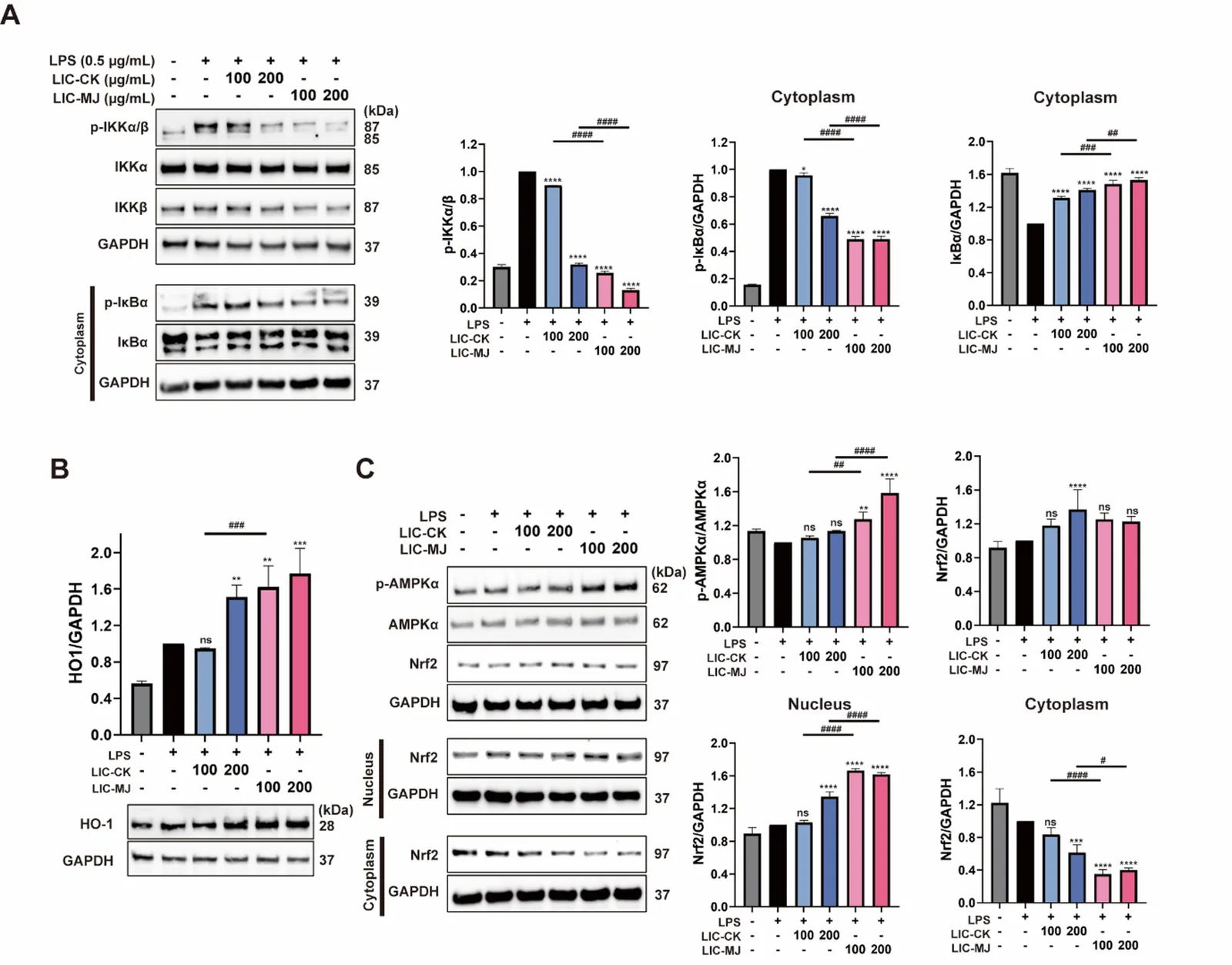
Effects of lavandin cell extracts on NF-κB and AMPK/Nrf2/HO-1 signaling pathways in LPS-stimulated RAW 264.7 cells. (**A**) RAW 264.7 cells were pretreated with LIC-CK or LIC-MJ for 1 h and stimulated with 0.5 µg/mL LPS for 15 min. Phosphorylated IKKα/β (p-IKKα/β) and total IKKα/IKKβ in whole-cell lysates, as well as phosphorylated IκBα (p-IκBα) and total IκBα in cytoplasmic fractions, were analyzed by Western blotting. (**B**, **C**) RAW 264.7 cells were pretreated with LIC-CK or LIC-MJ for 1 h and stimulated with 0.5 µg/mL LPS. (**B**) HO-1 protein expression was assessed after 12 h of LPS induction. (**C**) Phosphorylated AMPKα (p-AMPKα), total AMPKα, and Nrf2 in whole-cell lysates, together with nuclear translocation of Nrf2, were analyzed after 30 min of LPS induction. Quantitative analyses of Western blots were performed using ImageJ software. Data are means ± SD (*n* = 3). Statistical significance is indicated relative to the control. *, *p* < 0.05; **, *p* < 0.01; ***, *p* < 0.001; ****, *p* < 0.0001 vs. LPS group. #, *p* < 0.05; ##, *p* < 0.01; ###, *p* < 0.001; ####, *p* < 0.0001 vs. LIC-CK group

To further assess upstream regulation, we examined the IKK and IκB, which are key mediators of NF-κB activation. Under inflammatory stimulation, phosphorylation of IKK induces degradation of IκB, thereby releasing NF-κB to translocate into the nucleus and promote transcription of pro-inflammatory genes [11]. As shown in Fig. 7A, both LIC-CK and LIC-MJ inhibited the phosphorylation of IKKα/β and IκBα. Pretreatment with either extract also resulted in a dose-dependent increase in cytosolic IκBα protein levels, indicating stabilization of IκBα and effective inhibition of NF-κB activation. Specifically, phosphorylation of IKKα/β was reduced to 0.90- and 0.32-fold with LIC-CK (100 and 200 µg/mL) and to 0.26- and 0.13-fold with LIC-MJ (100 and 200 µg/mL), respectively. In parallel, cytosolic p-IκBα levels decreased to 0.96- and 0.66-fold with LIC-CK and to 0.49-fold with LIC-MJ, whereas total IκBα levels increased to 1.31- and 1.41-fold with LIC-CK and to 1.48- and 1.53-fold with LIC-MJ, further supporting inhibition of NF-κB signaling.

We next investigated whether lavandin cell extracts modulate antioxidant defense pathways. Upregulation of HO-1 is known to play a pivotal role in attenuating oxidative stress and inflammation [12]. As shown in Fig. 7B, Western blot analysis confirmed that lavandin cell extracts enhanced HO-1 protein expression. Specifically, HO-1 levels increased to 0.95- and 1.51-fold with LIC-CK (100 and 200 µg/mL) and to 1.62- and 1.77-fold with LIC-MJ (100 and 200 µg/mL), respectively, with LIC-MJ showing a stronger inductive effect. Additionally, to further elucidate the regulatory mechanism, we examined the expression of Nrf2, a key transcription factor that regulates HO-1 expression, and AMPK, which modulates Nrf2 phosphorylation and nuclear translocation [15]. As shown in Fig. 7C, both LIC-CK and LIC-MJ induced AMPKα phosphorylation and promoted Nrf2 nuclear translocation. Specifically, phosphorylation of AMPKα increased to 1.05- and 1.13-fold with LIC-CK (100 and 200 µg/mL) and to 1.27- and 1.58-fold with LIC-MJ (100 and 200 µg/mL), respectively. Total Nrf2 expression increased to 1.18- and 1.37-fold with LIC-CK and to 1.25- and 1.23-fold with LIC-MJ, while nuclear Nrf2 levels were markedly elevated to 1.03- and 1.35-fold with LIC-CK and to 1.66- and 1.62-fold with LIC-MJ. Conversely, cytosolic Nrf2 levels decreased to 0.83- and 0.61-fold with LIC-CK and to 0.35- and 0.40-fold with LIC-MJ, indicating enhanced nuclear translocation of Nrf2.

**Fig. 8:**
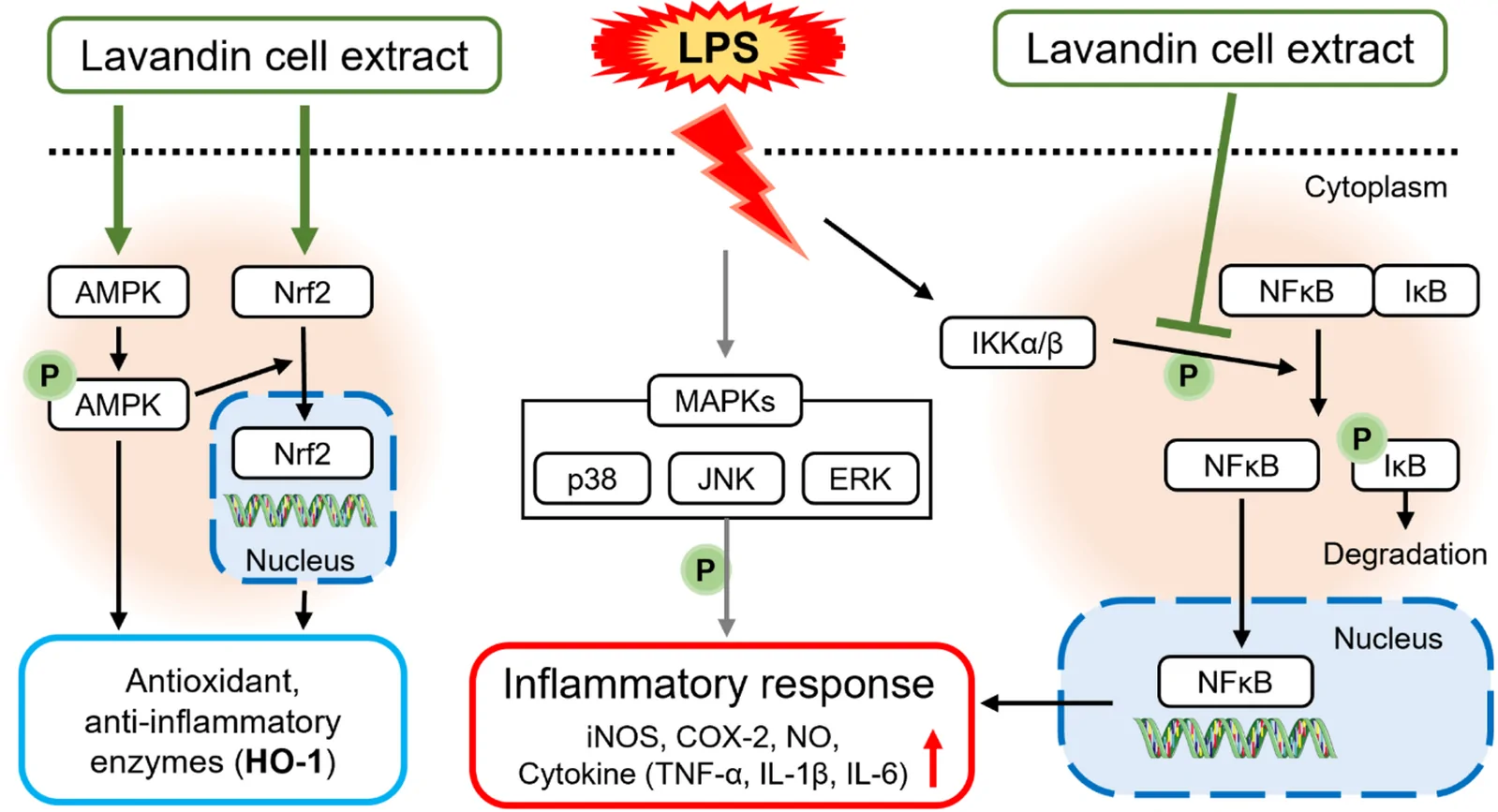
Schematic representation of the anti-inflammatory activities of lavandin cell extract in LPS-stimulated RAW 264.7 macrophages. Lavandin cell extract reduces NO and pro-inflammatory cytokine production and suppresses iNOS and COX-2 expression. These effects are mediated through inhibition of the IKK/IκB/NF-κB signaling pathway and activation of the AMPK/Nrf2/HO-1 pathway

Taken together, these results indicate that lavandin cell extracts exert anti-inflammatory effects through dual regulation: suppression of the IKK/IκB/NF-κB pathway and activation of the AMPK/Nrf2/HO-1 pathway. Across all assays, LIC-MJ consistently showed stronger activity than LIC-CK (Fig. 8).

## Discussion

Lavender is well known for its strong anti-inflammatory properties, largely attributed to its diverse repertoire of bioactive compounds, including linalool, phenolic acids, flavonoids, flavanols, coumarins, and triterpenes [24]. Among these, RA—a phenolic acid—has received particular attention due to its well-documented antioxidant and anti-inflammatory activities [8].

In our previous work, we successfully established cell suspension cultures of *L. angustifolia* and demonstrated that MJ elicitation significantly enhanced RA accumulation, which was correlated with notable antioxidant and skin-protective activities [4, 5]. Although these results highlighted the potential of lavender cell cultures as a platform for producing functional bioactive materials, further enhancement of RA productivity was considered necessary to improve scalability and broaden potential applications. To address this, the present study extended our approach to *L. intermedia* (lavandin), a naturally occurring hybrid known for its higher biomass and richer metabolite profile. Our results showed that lavandin cell cultures consistently produced greater amounts of RA under identical MJ treatment conditions compared to *L. angustifolia* (Figure S1), indicating that lavandin possesses an intrinsically higher biosynthetic capacity for RA production. This observation provided strong justification for focusing on lavandin not only as an alternative source but also as a more promising system for generating RA-enriched cell extracts with distinct biological activities.

Building on this rationale, we developed a lavandin cell extract enriched in RA using plant cell culture technology combined with MJ elicitation. To further characterize the chemical composition of the extracts, LC–MS metabolite profiling was performed for LIC-CK and LIC-MJ cell extracts (Figure S2, Table S3). Although the analysis was qualitative, differences in metabolite patterns were observed between the two extracts, and RA was detected as a prominent metabolite in LIC-MJ. This observation is consistent with the higher RA content quantified by HPLC, indicating that MJ elicitation enhances RA accumulation in lavandin cell cultures. Given the well-documented anti-inflammatory activity of RA, its enrichment may partly contribute to the stronger bioactivity of the LIC-MJ cell extract observed in this study. At the same time, the presence of multiple phenolic metabolites suggests that the biological activity of the extract may also involve combined or synergistic interactions among co-occurring compounds. The resulting extract, LIC-MJ, contained 5.9% RA, corresponding to RA concentrations of 5.9 µg/mL and 11.8 µg/mL at treatment doses of 100 µg/mL and 200 µg/mL, respectively. These concentrations fall within the range previously reported to elicit anti-inflammatory activity of pure RA, suggesting that RA present in the lavandin extract contributes significantly to the observed effects [25, 26].

The anti-inflammatory effects of RA have been demonstrated in a variety of inflammatory disease models [8]. In particular, RA inhibits the IKK/IκB/NF-κB signaling pathway in RAW 264.7 macrophages and other cell types, which is consistent with our findings [8, 26, 27]. By contrast, although RA has been reported to modulate MAPK signaling in certain contexts, our study did not reveal a clear inhibitory effect of lavandin cell extract on MAPK pathways. Indeed, relatively few studies have directly demonstrated MAPK inhibition by RA in RAW 264.7 macrophages, whereas substantial evidence supports its inhibitory role in NF-κB signaling [8, 26, 27]. Studies of *Lavandula* extracts generally demonstrate strong suppression of NF-κB signaling, with limited or selective effects on MAPK pathways. For example, *L. viridis* essential oil inhibited NF-κB–dependent iNOS expression, and lavender essential oil in THP-1 macrophages reduced NF-κB–driven cytokine production; however, neither study evaluated MAPK signaling [28, 29]. In a keratinocyte psoriasis model, *L. angustifolia* extract suppressed NF-κB–related genes and selectively reduced JNK expression without affecting ERK [24]. “Taikong blue” lavender oil study in HaCaT and RAW 264.7 cells reported strong reductions in NF-κB components with only moderate inhibition of p38 and JNK [30]. These observations support our results, indicating that lavandin cell extract primarily targets the NF-κB pathway, with little impact on MAPK activation. Further research, including dose–response studies, time-course analyses, and MAPK-specific inhibitor assays, will be required to clarify whether RA or lavandin cell extract can exert complementary effects on MAPK signaling in macrophages.

In addition to NF-κB suppression, our study demonstrated that LIC-MJ more effectively activated the AMPK/Nrf2/HO-1 pathway. This pathway is widely recognized as a central defense mechanism against oxidative stress and inflammation, contributing not only to cellular protection but also to systemic regulation of chronic inflammatory processes [15]. Although no prior studies have reported this effect in lavandin, extracts from other lavender species such as *L. angustifolia* and *L. multifida* have been shown to activate the AMPK or Nrf2/HO-1 axes, supporting the findings of the present study [31, 32]. Notably, RA is considered a key bioactive constituent involved in AMPK/Nrf2/HO-1 signaling, which likely explains the stronger pathway activation observed with LIC-MJ compared to LIC-CK [33, 34]. These findings underscore the broader therapeutic potential of RA-enriched lavandin cell extract, extending beyond macrophage-mediated inflammation to oxidative stress–related and chronic inflammatory diseases. Likewise, while several plant-derived extracts have been reported to modulate intracellular signaling pathways, the precise molecular mechanisms by which they act within cells remain incomDipletely understood [35]. This highlights the need for further mechanistic studies to clarify the molecular targets and interactions of lavandin cell extract.

Our findings demonstrated that the lavandin cell extract strongly inhibited the NF-κB signaling pathway, whereas its effect on MAPK activation was minimal. This suggests that the anti-inflammatory efficacy of the extract is primarily mediated through selective suppression of the IKK/IκB/NF-κB axis rather than broad inhibition of MAPK signaling. Selective inhibition of NF-κB offers potential therapeutic advantages over broader suppression of multiple signaling pathways. Because NF-κB is a central regulator of iNOS, COX-2, and numerous pro-inflammatory cytokines, its inhibition can effectively downregulate key inflammatory mediators while minimizing interference with other signaling cascades that are essential for normal cellular function [36, 37]. In contrast, indiscriminate inhibition of MAPK pathways may compromise cell survival, proliferation, and stress responses, raising the risk of undesirable side effects [38, 39]. Therefore, agents such as RA-enriched lavandin cell extract, which preferentially target the NF-κB axis while sparing MAPK signaling, may represent safer and more focused anti-inflammatory candidates with greater translational potential for chronic inflammatory diseases.

Beyond the mechanistic findings, this study demonstrates that elicitor-driven plant cell culture provides a reproducible platform for generating RA-enriched extracts with consistent anti-inflammatory activity. Although LIC-MJ is a complex extract, such systems are biologically relevant [40], and chromatographic analysis confirmed RA as the dominant component (Figure S2). To further support batch-to-batch consistency of the extracts, we evaluated independent cultures by measuring biomass accumulation, extract yield, and the concentration of RA as a marker compound, which showed comparable levels among batches (Table S4). While the contribution of individual constituents remains to be clarified, the dose-dependent and reproducible effects observed across multiple assays support the robustness of the bioactivity. Future studies focusing on the isolation of individual compounds and their mechanistic roles will further clarify these interactions. In parallel, microbial engineering has been explored for RA production [41]; however, these approaches serve different purposes, with microbial systems optimized for production efficiency and plant cell cultures enabling the production of phytochemically relevant extracts with validated biological activity.

This research uses rosmarinic acid (RA) content as a reference standard to analyze downstream proteins, including AMPK/Nrf2/HO-1 activation and IKK/IκB/NF-κB inhibition. The main reason is that the extracts are still a mixture, and it cannot be ruled out that the chemical composition is completely consistent. However, we have made every effort to use RA as a reference standard, and this study should have certain value in scientific field.

## Conclusions

This study demonstrated that cell suspension cultures of *L. intermedia* (lavandin), when combined with MJ elicitation, can produce RA-enriched cell extracts with potent anti-inflammatory activity. Compared with *L. angustifolia* cell cultures, lavandin cell cultures exhibited a greater capacity for RA accumulation, and MJ treatment further enhanced RA production through transcriptional activation of RA biosynthetic genes. The MJ-treated lavandin cell extracts significantly reduced NO production, pro-inflammatory cytokine release, and the expression of iNOS and COX-2 in LPS-stimulated RAW 264.7 macrophages. Mechanistic studies revealed that these effects were primarily mediated by suppression of the IKK/IκB/NF-κB pathway, exerted with minimal influence on MAPK signaling, and were accompanied by activation of the AMPK/Nrf2/HO-1 axis.

Collectively, these findings highlight the strong anti-inflammatory activity of RA-enriched lavandin cell extracts and underscore the potential of lavandin cell cultures as a promising and sustainable platform for generating functional biomaterials. Although further in vivo validation and detailed mechanistic studies are warranted, our results provide a solid scientific basis for the potential application of lavandin cell extracts in the development of therapeutic and cosmeceutical agents targeting oxidative stress–related and chronic inflammatory diseases. 

## Supplementary Information

Supplementary Material 1: Figure S1-2, Table S1-4; Figure S1. Comparative analysis of RA production in cell suspension cultures of lavender (*L. angustifolia*) and lavandin (*L. intermedia*) following treatment with 100 µM MJ; Figure S2. Representative BPI chromatograms of lavandin cell extracts; Table S1. Primer sequences used for RT-qPCR; Table S2. Primary and secondary antibodies used in Western blot analysis; Table S3. LC-MS results for metabolites identified in LIC-MJ and LIC-CK; Table S4. Batch-to-batch comparison of biomass, 80% methanol extract yield, and RA contents in independent lavandin cell suspension cultures.

Supplementary Material 2. Original Western blot images.

## Data Availability

The data that support the findings of this study are available from the corresponding author, Cha Young Kim, upon reasonable request.

## Abbreviations

ABA: Abscisic acid
AAT1: Acyltransferase 1
AMPK: AMP-activated protein kinase
AsA: L-ascorbic acid
BAP: 6-benzylaminopurine
C4H: Cinnamate 4-hydroxylase
COX-2: Cyclooxygenase-2
CYP450: Cytochrome P450 monooxygenase
DMEM: Dulbecco’s Modified Eagle Medium
DMSO: Dimethyl sulfoxide
DPPH: 2,2-diphenyl-1-picrylhydrazyl
DW: Dry weight
ELISA: Enzyme-linked immunosorbent assay
ERK: Extracellular signal-regulated kinase
ET: Ethephon
FBS: Fetal bovine serum
HO-1: Heme oxygenase-1
HPPR: Hydroxyphenylpyruvate reductase
HPLC: High-performance liquid chromatography
IKBα: Inhibitor of κB alpha
IKK: Inhibitor of κB kinase
IL-1β: Interleukin-1 beta
IL-6: Interleukin-6
iNOS: inducible nitric oxide synthase
JNK: c-Jun N-terminal kinase
LIC-CK: Lavandin cell extract from untreated cells
LIC-MJ: Lavandin cell extract from methyl jasmonate-treated cells
LPS: Lipopolysaccharide
MAPK: Mitogen-activated protein kinase
MJ: Methyl jasmonate
MS: Murashige and Skoog
NF-κB: Nuclear factor kappa B
NO: Nitric oxide
Nrf2: Nuclear factor erythroid 2-related factor 2
PAL: Phenylalanine ammonia-lyase
PGE2: Prostaglandin E2
PTFE: Polytetrafluoroethylene
RA: Rosmarinic acid
RAS: Rosmarinic acid synthase
RT-qPCR: Reverse transcription quantitative polymerase chain reaction
SA: Salicylic acid
SDS-PAGE: Sodium dodecyl sulfate-polyacrylamide gel electrophoresis
TAT: Tyrosine aminotransferase
TNF-α: Tumor necrosis factor alpha
4CL: 4-coumarate: CoA ligase
