# Supplementary material for: Lavandin cell extract enriched in rosmarinic acid attenuates inflammation via AMPK/Nrf2/HO-1 activation and IKK/IκB/NF-κB inhibition
Source: BMC Plant Biol. 2026 Jun 27;26:1582. doi: 10.1186/s12870-026-09361-5 (PMC13573341; doi:10.1186/s12870-026-09361-5)
Supplement: Supplementary file 1 [file 12870_2026_9361_MOESM1_ESM.docx]

**Supplementary Material 1: Figure S1-2, Table S1-S4**

**Lavandin cell extract enriched in rosmarinic acid attenuates inflammation via AMPK/Nrf2/HO-1 activation and IKK/IκB/NF-κB inhibition**

Gyeongchan Jeon ^1, †^, Bo Ryeong Kim ^1, 2, †^, Yu Jeong Jeong ^1^, Soyoung Kim ^1^, Jiyoung Lee ^1^, Ok Ran Lee ^2^, Hyung Jae Jeong ^3^, Jae Cheol Jeong ^1^, Cha Young Kim ^1, *^

*^1^ Biological Resource Center, Korea Research Institute of Bioscience Biotechnology (KRIBB), Republic of Korea, Jeongeup 56212, Republic of Korea*

*^2^ Department of Applied Plant Science, College of Agriculture and Life Sciences, Chonnam National University, Gwangju 61186, Republic of Korea*

*^3^* *Functional Biomaterial Research Center, Korea Research Institute of Bioscience Biotechnology (KRIBB), Republic of Korea, Jeongeup 56212, Republic of Korea*

^†^ Gyeongchan Jeon and Bo Ryeong Kim authors contributed equally to this work.

*Corresponding author. Cha Young Kim (E-mail: kimcy@kribb.re.kr)

Dr. Cha Young Kim

Biological Resource Center,

Korea Research Institute of Bioscience and Biotechnology (KRIBB),

181 Ipsin-gil, Jeongeup-si, Jeonbuk 56212, Republic of Korea

**Contents**

**Figure S1**. Comparative analysis of RA production in cell suspension cultures of lavender (*L. angustifolia*) and lavandin (*L. intermedia*) following treatment with 100 µM MJ

**Figure S2.** Representative BPI chromatograms of lavandin cell extracts

**Table S1**. Primer sequences used for RT-qPCR

**Table S2**. Primary and secondary antibodies used in Western blot analysis

**Table S3.** LC-MS results for metabolites identified in LIC-MJ and LIC-CK

**Table S4**. Batch-to-batch comparison of biomass, 80% methanol extract yield, and RA contents in independent lavandin cell suspension cultures

**Figure S1**. Comparative analysis of rosmarinic acid (RA) production in cell suspension cultures of lavender (*L. angustifolia*) and lavandin (*L. intermedia*) following treatment with 100 µM methyl jasmonate (MJ). Cells were pre-cultured for 7 days, followed by treatment with 100 μM MJ for 5 days. The harvested samples were dried, extracted with 80% methanol, and analyzed by HPLC to compare RA contents.

**Figure S2.** Representative BPI chromatograms of lavandin cell extracts. UPLC-Q-TOF-MS analysis was performed in (A) negative and (B) positive ESI modes. QC, quality control; LIC-MJ, MJ-treated cell extract; LIC-CK, untreated cell extract. Separation was achieved on an ACQUITY UPLC HSS T3 column (100 × 2.1 mm, 1.8 µm) at 40°C with a 0.35 mL/min gradient of 0.1% formic acid in water and acetonitrile. MS conditions: scan range *m/z* 50–1200; capillary voltage 2.0 kV; collision energy 20–50 eV. Peaks are numbered by identification order.

**Table S1**. Primary and secondary antibodies used in Western blot analysis

| **Target protein (clone)** | **Host** | **Supplier (Company, country)** | **Catalog No.** |
| --- | --- | --- | --- |
| **Primary antibodies** | | | |
| GAPDH (14C10) | Rabbit | Cell Signaling, USA | #2118 |
| COX-2 (D5H5 XP®) | Rabbit | Cell Signaling, USA | #12282 |
| NOS2/iNOS | Mouse | Santa Cruz, USA | sc-7271 |
| Phospho-NF-κB p65 | Rabbit | Cell Signaling, USA | #3033 |
| NF-κB p65 | Rabbit | Cell Signaling, USA | #8242 |
| Phospho-p38 MAPK | Rabbit | Cell Signaling, USA | #4511 |
| p38 MAPK | Rabbit | Cell Signaling, USA | #8690 |
| Phospho-SAPK/JNK | Rabbit | Cell Signaling, USA | #4668 |
| SAPK/JNK | Rabbit | Cell Signaling, USA | #9252 |
| Phospho-p44/42 MAPK (ERK1/2) | Rabbit | Cell Signaling, USA | #4370 |
| p44/42 MAPK (ERK1/2) | Rabbit | Cell Signaling, USA | #4695 |
| Phospho-IKKα/β (Ser176/180) (16A6) | Rabbit | Cell Signaling, USA | #2697 |
| IKKα (D3W6N) | Rabbit | Cell Signaling, USA | #61294 |
| IKKβ (D30C6) | Rabbit | Cell Signaling, USA | #8943 |
| Phospho-IκBα | Rabbit | Cell Signaling, USA | #2859 |
| IκBα | Rabbit | Cell Signaling, USA | #4812 |
| HO-1 | Rabbit | Cell Signaling, USA | #43966 |
| Nrf2 | Rabbit | Cell Signaling, USA | #12721 |
| Phospho-AMPKα | Rabbit | Cell Signaling, USA | #2535 |
| AMPKα | Rabbit | Cell Signaling, USA | #2532 |
| **Secondary antibodies** | | | |
| Anti-rabbit IgG, HRP-linked | Goat | Cell Signaling, USA | #7074 |
| Anti-mouse IgG, HRP-linked | Goat | Cell Signaling, USA | #7076 |

**Table S2**. Primer sequences used for RT-qPCR

| Genes | Primer Sequence (5’-3’) |
| --- | --- |
| *LaACTIN* | F : TGCTCAGTGGTGGATCGACTATG |
|  | R : ACCACCTTAATCTTCATGCTGCTTG |
| *LaTUBULIN* | F : CCACAATCAAGACCAAGAGGACAATC |
|  | R : CGCCAGGGACCACGGTTG |
| *LaPAL* | F : GAAGCCTTCGAAGAGGAGTTAAA |
|  | R : ACCGAGCTCCTCTCTTATGAACT |
| *LaC4H* | F : TGATCAAGGAAACTCTTCGTCTC |
|  | R : CTCCGGTCTAAACTCTTCAGGAT |
| *La4CL* | F : AGGCTGGTCCAGTACTATCAATG |
|  | R : TAGCAGTAGCCTCAGGATCATTC |
| *LaTAT* | F : CGAGAAGGGATGGGAGGTTG |
|  | R : TCCCAAGTCTCTTTGCGGTC |
| *LaHPPR* | F : CACGCTAAAGCACGAAACTATCT |
|  | R : TTCGCTCTCTATCGTTACTGGAC |
| *LaAAT1* | F : TTACGCTTCACAAGCAGCAC |
|  | R : CAAAGTCACCAAACGCAGAA |
| *LaCYP450* | F : AGGCTGGGATCACATCAAAG |
|  | R : TTTTCCCCACCAAGTTTCTG |
| *GAPDH* | F : ACTCCACTCACGGCAAATTC |
|  | R : CCCTGTTGCTGTAGCCGTAT |
| *TNF-α* | F : CCACGCTCTTCTGTCTACTGAAC |
|  | R : ACAGGCTTGTCACTCGAA |
| *IL-1β* | F : TGCCACCTTTTGACAGTGATGA |
|  | R : TGTGCTGCTGCGAGATTTGA |
| *IL-6* | F : CCGGAGAGGAGACTTCACAG |
|  | R : TCCACGATTTCCCAGAGAAC |
| *iNOS* | F : GTTCTCAGCCCAACAATACAAGA |
|  | R : GTGGACGGGTCGATGTCAC |
| *COX-2* | F : GTATGAGCACAGGATTTGACCA |
|  | R : TGTTCACAATGTTCCAGACTCC |

**Table S3.** List of metabolites identified in LIC-MJ and LIC-CK cell extracts by UPLC-Q-TOF-MS

| **No.** | **Metabolite** | **RT**  **(min)** | **Adduct** | **Precursor ion (*m/z*)** | | **Mass error (ppm)** | **Chemical**  **formula** | **Fragment ion (*m/z*)** | **Fold change (LIC-MJ**  **/LIC-CK)** |
| --- | --- | --- | --- | --- | --- | --- | --- | --- | --- |
|  |  |  |  | Calculated mass | Observed mass |  |  |  |  |
| 1 | Gluconic acid | 0.74 | [M-H]^-^ | 195.0505 | 195.0512 | 3.6 | C_6_H_12_O_7_ | 159.0304, 129.0195 | 1.39^***^ |
| 2 | Sucrose | 0.81 | [M-H]^-^ | 341.1084 | 341.1089 | 1.5 | C_12_H_22_O_11_ | 179.0564, 161.0457 | 0.67^***^ |
| 3 | Ethyl glucoside | 1.60 | [M+Na]^+^ | 231.0845 | 231.0850 | 2.2 | C_8_H_16_O_6_ | 127.0403, 145.0503 | 0.66^***^ |
| 4 | Adenosine | 2.20 | [M+H]^+^ | 268.1046 | 268.1051 | 1.9 | C_10_H_13_N_5_O_4_ | 136.0628 | 0.76 |
| 5 | Ethyl glucosyl-fructoside | 2.38 | [M-H]^-^ | 369.1397 | 369.1402 | 1.4 | C_14_H_26_O_11_ | 207.0876, 161.0456 | 0.48^***^ |
| 6 | Ethyl glucoside derivative | 3.15 | [M-H]^-^ | 339.1291 | 339.1299 | 2.4 | C_13_H_24_O_10_ | 207.0878, 161.0452 | 0.54^***^ |
| 7 | Danshensu | 4.35 | [M-H]^-^ | 197.0450 | 197.0458 | 4.1 | C_9_H_10_O_5_ | 179.0349, 135.0457, 123.0445 | 19.76^***^ |
| 8 | Glucosyl-hydroxymethyl-butenoic acid derivative | 4.52 | [M-H]^-^ | 293.0873 | 293.0883 | 3.4 | C_11_H_18_O_9_ | 249.0981, 207.0874 | 0.24^***^ |
| 9 | Vanillic acid glucoside | 4.82 | [M-H]^-^ | 329.0873 | 329.0881 | 2.4 | C_14_H_18_O_9_ | 167.0354, 152.0120, 123.0456, 108.0220 | 1.29^***^ |
| 10 | Vanilloloside | 5.03 | [M-H]^-^ | 315.1080 | 315.1088 | 2.5 | C_14_H_20_O_8_ | 153.0558, 123.0452 | 1.63^***^ |
| 11 | Monoallyl sucrose | 5.37 | [M-H]^-^ | 381.1397 | 381.1406 | 2.4 | C_15_H_26_O_11_ | 339.1294, 299.0777, 207.0875, 161.0455 | 0.47^***^ |
| 12 | Caffeic acid | 6.88 | [M-H]^-^ | 179.0344 | 179.0353 | 5.0 | C_9_H_8_O_4_ | 135.0454 | 0.56^**^ |
| 13 | Sucrose benzoate | 7.32 | [M+Na]^+^ | 469.1322 | 469.1333 | 2.3 | C_19_H_26_O_12_ | 307.0795, 203.0538, 185.0424 | 0.03^***^ |
| 14 | Sibiricose A5 | 7.47 | [M-H]^-^ | 517.1557 | 517.1570 | 2.5 | C_29_H_30_O_14_ | 337.0930, 193.0508, 175.0401, 160.0159 | 0.49^***^ |
| 15 | Sibiricose A6 | 7.61 | [M+Na]^+^ | 571.1639 | 571.1650 | 1.9 | C_23_H_32_O_15_ | 409.1124, 207.0662, 175.0396, 147.0455, 119.0498 | 0.12^***^ |
| 16 | lithospermate-9'-*O*-glucoside | 8.03 | [M-H]^-^ | 699.1561 | 699.1570 | 1.3 | C_33_H_32_O_17_ | 655.1658, 457.1142, 295.0611 | 1.32^*^ |
| 17 | Salvianolic acid J | 8.77 | [M-H]^-^ | 537.1033 | 537.1042 | 1.7 | C_27_H_22_O_12_ | 295.0616, 197.0464, 185.0256, 179.0350, 161.0250 | 1.76^***^ |
| 18 | Feruloyltyramine derivative | 9.07 | [M+H]^+^ | 476.1934 | 476.1935 | 0.2 | C_25_H_25_N_5_O_5_ | 177.0554, 145.0291, 121.0658, 117.0353 | 0.75^*^ |
| 19 | Salviaflaside | 9.15 | [M-H]^-^ | 521.1295 | 521.1306 | 2.1 | C_24_H_26_O_13_ | 359.0980, 197.0456, 179.0347, 161.0251 | 0.37^***^ |
| 20 | Salvinoside | 9.33 | [M-H]^-^ | 879.1984 | 879.1993 | 1.0 | C_42_H_40_O_21_ | 681.1463, 519.0955, 501.1042, 483.0935, 457.1143, 295. 0614, 197.0457, 185.0246, 179.0352, 173.0232, 161.0253, 135.0455 | 1.71^***^ |
| 21 | Salvianolic acid derivative | 9.63 | [M-H]^-^ | 735.1561 | 735.1570 | 1.2 | C_36_H_32_O_17_ | 537.1041, 519.0935, 359.0773, 321.0388, 295.0610 | 2.29^***^ |
| 22 | Salvianolic acid E | 9.85 | [M-H]^-^ | 717.1456 | 717.1465 | 1.3 | C_36_H_30_O_16_ | 537.1041, 519.0936, 339.0512, 321.0406, 295.0613 | 1.88^***^ |
| 23 | Rosmarinic acid | 10.25 | [M-H]^-^ | 359.0767 | 359.0771 | 1.1 | C_18_H_16_O_8_ | 197.0456, 179.0351, 161.0246 | 1.57^***^ |
| 24 | Rosmarinic acid methylester | 11.64 | [M-H]^-^ | 373.0923 | 373.0930 | 1.9 | C_19_H_18_O_8_ | 179.0350, 135.0353 | 2.73^***^ |
| 25 | Methoxycoumarin | 11.64 | [M+H]^+^ | 177.0552 | 177.0552 | 0.0 | C_10_H_8_O_3_ | 149.0613, 134.0370, 117.0346 | 2.78^***^ |
| 26 | Triterpene saponin 1 | 13.28 | [M-H]^-^ | 983.4488 | 983.4505 | 1.7 | C_48_H_72_O_21_ | 895.4704, 853.4585, 835.4479, 487.3431 | 1.52^***^ |
| 27 | Salvianolic F | 13.64 | [M-H]^-^ | 313.0712 | 313.0719 | 2.2 | C_17_H_14_O_6_ | 313.0722, 161.0246 | 0.84 |
| 28 | Unknown phenolic compound | 14.81 | [M-H]^-^ | 897.2665 | 897.2675 | 1.1 | C_40_H_50_O_23_ | 809.2864, 767.2763, 749.2653, 667.3330, 625.3226, 555.3291, 487.3426, 463.2712 | 1.39^***^ |
| 29 | Unknown compound | 15.23 | [M-H]^-^ | 797.3232 | 797.3249 | 2.1 | C_38_H_54_O_18_ | 709.3441, 667.3337, 649.3226, 625.3224, 607.3125, 589.3016, 547.2888, 505.2809,487.3423, 463.2702, 445.2601 | 0.41^***^ |
| 30 | Triterpene saponin 2 | 16.20 | [M+H-H_2_O]^+^ | 469.3318 | 469.3333 | 3.2 | C_30_H_46_O_5_ | 451.3229, 433.3123, 423.3274, 405.3172 | 1.34^***^ |
| 31 | Triterpene saponin 3 | 16.28 | [M+H-H_2_O]^+^ | 471.3474 | 471.3491 | 3.6 | C_30_H_48_O_5_ | 453.3386, 435.3279, 425.3436, 407.3329 | 1.32^***^ |
| 32 | Triterpene saponin 4 | 16.36 | [M+H-H_2_O]^+^ | 471.3474 | 471.3489 | 3.2 | C_30_H_48_O_5_ | 453.3386, 435.3278, 425.3434, 407.3333 | 1.24^***^ |
| 33 | Triterpene saponin 5 | 16.74 | [M+H-2H_2_O]^+^ | 453.3369 | 453.3386 | 3.7 | C_30_H_48_O_5_ | 435.3279, 425.3428, 407.3327 | 0.86^***^ |
| 34 | Triterpene saponin 6 | 17.03 | [M+H]^+^ | 635.3948 | 635.3967 | 3.1 | C_39_H_54_O_7_ | 453.3387, 435.3283, 407.3329 | 0.75^*^ |
| 35 | Triterpene saponin 7 | 17.12 | [M+H]^+^ | 665.4053 | 665.4064 | 1.7 | C_40_H_56_O_8_ | 453.3384, 435.3282, 407.3332 | 1.10^**^ |
| 36 | Triterpene saponin 8 | 17.31 | [M+H]^+^ | 473.3631 | 473.3647 | 3.4 | C_30_H_48_O_4_ | 455.3539, 437.3434, 409.3483 | 1.22^***^ |
| 37 | Triterpene saponin 9 | 18.04 | [M+H]^+^ | 619.3999 | 619.4019 | 3.2 | C_39_H_54_O_6_ | 455.3533, 437.3438, 409.3482, 391.3378 | 0.70^***^ |
| 38 | Triterpene saponin 10 | 18.14 | [M+H]^+^ | 469.3318 | 469.3334 | 3.4 | C_30_H_44_O_4_ | 451.3229, 433.3128, 423.3278, 405.3171 | 4.60^***^ |
| 39 | Triterpene saponin 11 | 18.25 | [M+H]^+^ | 649.4104 | 649.4121 | 2.6 | C_40_H_56_O_7_ | 437.3440, 411.3280 | 1.14^*^ |
| 40 | Triterpene saponin 12 | 18.97 | [M+H-H_2_O]^+^ | 439.3576 | 439.3598 | 5.0 | C_30_H_48_O_2_ | 411.3635, 393.3527 | 1.28^***^ |
| 41 | Triterpene saponin 13 | 19.13 | [M+H-H_2_O]^+^ | 439.3576 | 439.3596 | 4.6 | C_30_H_48_O_2_ | 411.3652, 393.3520 | 1.30^***^ |

**Table S4**. Batch-to-batch comparison of biomass, 80% methanol extract yield, and RA contents in independent lavandin cell suspension cultures (20 mL, n = 3 independent cultures). LIC-CK was cultured for 12 days, and LIC-MJ was pre-cultured for 7 days followed by 5 days of MJ elicitation. Samples were harvested, dried, extracted with 80% methanol, and analyzed by HPLC for RA quantification.

|  | Biomass  (mg DW) | 80% MtOH extract  (mg DW) | RA contents  (mg) |
| --- | --- | --- | --- |
| LIC-CK | 355.7 ± 14.6 | 47.0 ± 3.6 | 1.67 ± 0.15 |
| LIC-MJ | 352.0 ± 24.8 | 40.3 ± 1.7 | 8.25 ± 0.83 |
