# Supplementary material for: Lavandin cell extract enriched in rosmarinic acid attenuates inflammation via AMPK/Nrf2/HO-1 activation and IKK/IκB/NF-κB inhibition
Source: BMC Plant Biol. 2026 Jun 27;26:1582. doi: 10.1186/s12870-026-09361-5 (PMC13573341; doi:10.1186/s12870-026-09361-5)
Supplement: Supplementary file 2 [file 12870_2026_9361_MOESM2_ESM.docx]

**Supplementary Material 2: Original western blot images**

**Lavandin cell extract enriched in rosmarinic acid attenuates inflammation via AMPK/Nrf2/HO-1 activation and IKK/IκB/NF-κB inhibition**

Gyeongchan Jeon ^1, †^, Bo Ryeong Kim ^1, 2, †^, Yu Jeong Jeong ^1^, Soyoung Kim ^1^, Jiyoung Lee ^1^, Ok Ran Lee ^2^, Hyung Jae Jeong ^3^, Jae Cheol Jeong ^1^, Cha Young Kim ^1, *^

*^1^ Biological Resource Center, Korea Research Institute of Bioscience Biotechnology (KRIBB), Republic of Korea, Jeongeup 56212, Republic of Korea*

*^2^ Department of Applied Plant Science, College of Agriculture and Life Sciences, Chonnam National University, Gwangju 61186, Republic of Korea*

*^3^* *Functional Biomaterial Research Center, Korea Research Institute of Bioscience Biotechnology (KRIBB), Republic of Korea, Jeongeup 56212, Republic of Korea*

^†^ Gyeongchan Jeon and Bo Ryeong Kim authors contributed equally to this work.

*Corresponding author. Cha Young Kim (E-mail: kimcy@kribb.re.kr)

Dr. Cha Young Kim

Biological Resource Center,

Korea Research Institute of Bioscience and Biotechnology (KRIBB),

181 Ipsin-gil, Jeongeup-si, Jeonbuk 56212, Republic of Korea

**Used ladder in western blot analysis (PM2700 ExcelBand™ 3-color Broad Range Protein Marker)**

**Western blot raw images of Figure 6A**

**Western blot raw images of Figure 6B**

**Western blot raw images of Figure 6C**

**Western blot raw images of Figure 7A**

**Western blot raw images of Figure 7B**

**Western blot raw images of Figure 7C**
